# Tobacco Use, Nicotine Dependence, and Cessation Methods in US Adults With Psychosis

**DOI:** 10.1001/jamanetworkopen.2023.4995

**Published:** 2023-03-28

**Authors:** Beth Han, Ther W. Aung, Nora D. Volkow, Marushka L. Silveira, Heather L. Kimmel, Carlos Blanco, Wilson M. Compton

**Affiliations:** 1 National Institute on Drug Abuse, National Institutes of Health, Bethesda, Maryland; 2 National Institute of Dental and Craniofacial Research, National Institutes of Health, Bethesda, Maryland; 3 Kelly Government Solutions, Rockville, Maryland

## Abstract

**Importance:**

Adults with psychotic disorders have high premature mortality, partly due to the high prevalence of smoking in this population. Yet recent data are lacking on tobacco product use among US adults with a history of psychosis.

**Objective:**

To examine the sociodemographic characteristics and behavioral health status; types of tobacco products used; prevalence of use by age, sex, and race and ethnicity; and nicotine dependence severity and smoking cessation methods among community-dwelling adults with vs without psychosis.

**Design, Setting, and Participants:**

This cross-sectional study analyzed nationally representative, self-reported, cross-sectional data of adults (aged ≥18 years) who participated in the Wave 5 survey (conducted from December 2018 to November 2019) of the Population Assessment of Tobacco and Health (PATH) Study. Data analyses were conducted between September 2021 and October 2022.

**Exposure:**

PATH Study respondents were classified as having lifetime psychosis if they answered *yes* to whether they had ever received from a clinician (eg, physician, therapist, or other mental health professional) a diagnosis of schizophrenia, schizoaffective disorder, psychosis, or psychotic illness or episode.

**Main Outcomes and Measures:**

Use of any and major types of tobacco products, severity of nicotine dependence, and cessation methods.

**Results:**

Among the 29 045 community-dwelling adults who participated in the PATH Study (weighted median [IQR] age, 30.0 [22.0-50.0] years; weighted percentage estimates: 14 976 females (51.5%); 16.0% Hispanic, 11.1% non-Hispanic Black, 65.0% non-Hispanic White, and 8.0% non-Hispanic other race and ethnicity [American Indian or Alaska Native, Asian, Native Hawaiian or other Pacific Islander, and more than 1 race]), 2.9% (95% CI, 2.62%-3.10%) reported receiving a lifetime psychosis diagnosis. Compared with those without psychosis, people with psychosis had a higher adjusted prevalence of past-month any tobacco use (41.3% vs 27.7%; adjusted risk ratio [RR], 1.49 [95% CI, 1.36-1.63]) as well as cigarette smoking, e-cigarette use, and other tobacco product use overall and in most examined subgroups; they also had a higher past-month prevalence of dual cigarette and e-cigarette use (13.5% vs 10.1%; *P* = .02), polycombustible tobacco use (12.1% vs 8.6%; *P* = .007), and polycombustible and noncombustible tobacco use (22.1% vs 12.4%; *P* < .001). Among adults with past-month cigarette use, those with vs without psychosis had a higher adjusted mean nicotine dependence scores overall (54.6 vs 49.5; *P* < .001) and within the 45-years-or-older (61.7 vs 54.9; *P* = .002), female (56.9 vs 49.8; *P* = .001), Hispanic (53.7 vs 40.0; *P* = .01), and Black (53.4 vs 46.0; *P* = .005) groups. They were also more likely to make a quit attempt (60.0% vs 54.1%; adjusted RR, 1.11 [95% CI, 1.01-1.21]) and use counseling, a quitline, or a support group for tobacco cessation (5.6% vs 2.5%; adjusted RR, 2.25 [95% CI, 1.21-3.30]).

**Conclusions and Relevance:**

In this study, the high prevalence of tobacco use, polytobacco use, and making a quit attempt as well as the severity of nicotine dependence among community-dwelling adults with a history of psychosis highlighted the urgency for tailored tobacco cessation interventions for this population. Such strategies must be evidence-based and age, sex, and race and ethnicity appropriate.

## Key Points

**Question:**

Do prevalence of tobacco product use, severity of nicotine dependence, and cessation methods among community-dwelling adults differ between persons with and without psychosis?

**Findings:**

In this cross-sectional study of 29 045 adult participants in the Population Assessment of Tobacco and Health Study, those with psychosis vs those without psychosis had a higher past-month prevalence of any tobacco, cigarette, e-cigarette, and other tobacco product use; a higher past-month prevalence of polytobacco use; higher nicotine dependence scores; and a higher past-year prevalence of making a quit attempt.

**Meaning:**

Findings of this study suggest that tobacco cessation interventions tailored for community-dwelling adults with psychosis are urgently needed.

## Teaser

This cross-sectional study assesses the self-reported use of tobacco products and other substances and related behaviors by adults with and without a history of schizophrenia, schizoaffective disorder, psychosis, or psychotic illness.

## Introduction

Studies have demonstrated that tobacco use is more prevalent among individuals with psychosis than in the general population.^1,2,3,4^ Based on nationally representative data collected in the US between 2004 and 2005, one study reported that 49.8% of adults with lifetime psychotic disorders or psychosis smoked cigarettes in the previous month.^5^ That study did not examine the prevalence of the full range of currently available tobacco products beyond cigarettes, particularly electronic nicotine delivery systems (ie, e-cigarettes).^5^ Yet e-cigarettes may represent a means of tobacco use initiation or a tobacco harm-reduction approach, especially among people with psychosis who are unable to quit using conventional smoking cessation approaches.^6^ Among outpatients with daily tobacco use and psychosis who were psychiatrically stable from 2013 to 2015 and completed a 6-month follow-up, almost one-half ever used e-cigarettes, and researchers highlighted the importance of incorporating e-cigarette use in tobacco use interventions.^7^

Moreover, in the US, tobacco use has been declining in recent years among persons with mental illness,^8^ and tobacco use prevention and control interventions have been increasing for this population.^1,2,4,8,9,10,11^ Nicotine replacement therapy (NRT) and counseling help people with psychosis who smoke cigarettes to quit tobacco use.^10^ The effectiveness and safety of pharmacotherapy also have been demonstrated for people with nicotine dependence and psychosis.^1,2,4^ Thus, an updated study is needed to estimate the prevalence of a comprehensive range of tobacco products and cessation methods using data from a nationally representative, community-dwelling adult sample with or without psychosis.

Although in the US nicotine dependence severity is high among inpatients with psychosis who smoke cigarettes,^12^ to our knowledge, no nationally representative studies have evaluated the severity of nicotine dependence and cessation methods (eg, e-cigarette use, NRT, counseling, and prescription medication) among community-dwelling individuals with or without psychosis who smoke. Additionally, although the age, sex, and racial and ethnic differences in the prevalence of tobacco use are well documented,^13,14,15,16,17^ little is known about the differences in tobacco use outcomes (for key sociodemographic variables) between those with and those without lifetime psychosis. Adjusting for differences in sociodemographic characteristics^13,14,15,16,17^ and behavioral conditions (eg, cannabis and other substance use^18^ and comorbid psychiatric problems^5^) is necessary to reduce potential confounders when examining age-, sex-, and race and ethnicity–specific differences in tobacco use–related outcomes between populations with or without psychosis.

To fill these gaps in the literature and inform tobacco cessation interventions for people with psychosis, we used recent nationally representative data to examine the differences between community-dwelling adults with and without lifetime psychosis with regard to (1) sociodemographic characteristics and behavioral health status; (2) distributions of types of tobacco products used; (3) prevalence of any tobacco use, cigarette use, e-cigarette use, and any other tobacco product use overall and by age, sex, and race and ethnicity; and (4) nicotine dependence severity and prevalence of smoking cessation methods used (among cigarette smokers).

## Methods

### Data Sources

This cross-sectional study analyzed restricted-use data on adults aged 18 or older who participated in the Wave 5 survey of the Population Assessment of Tobacco and Health (PATH) Study that was conducted from December 2018 to November 2019. The PATH Study is an ongoing US-based cohort study by the National Institute on Drug Abuse of the National Institutes of Health in partnership with the Center for Tobacco Products of the US Food and Drug Administration. Data collection for the PATH Study is conducted by Westat. The Westat Institutional Review Board approved the present study. All adult participants provided written informed consent. We followed the Strengthening the Reporting of Observational Studies in Epidemiology (STROBE) reporting guideline.

In the PATH Study, multistage, address-based, area-probability sampling and audio computer-assisted self-interviews were used to collect nationally representative self-reported data on tobacco use patterns and associated health behaviors. Weighted estimates, using Wave 5 single-wave weights, represented the US civilian, noninstitutionalized adult population. The weighted interview response rate at Wave 5 was 88.0% for adults. Additional details about the PATH Study are provided elsewhere.^19^

### Measures

Starting in Wave 4, survey respondents were asked whether they had ever received from a clinician (eg, physician, therapist, or other mental health professional) a diagnosis of schizophrenia, schizoaffective disorder, psychosis, or psychotic illness or episode. In Wave 5, respondents were asked the same question if they had not answered it in the previous survey; otherwise, respondents were asked about receiving such a diagnosis in the past 12 months. Respondents were classified as having lifetime psychosis if they answered *yes* to having ever had a diagnosis or a past-year diagnosis. These survey questions were adapted from the National Epidemiologic Survey on Alcohol and Related Conditions-III (NESARC-III) instrument, which collects self-reported data on health professional–diagnosed schizophrenia or a psychotic illness or episode.^20^ The NESARC-III instrument for schizophrenia has been widely used in peer-reviewed publications,^21,22,23^ and a similar self-report method has been evaluated^24^ and was found to have rates that were consistent with clinically diagnosed prevalence rates reported in the literature.^25,26^ Moreover, similar prevalence rates have been found across different cultures and countries.^26,27^

All other outcome measures and covariates in this cross-sectional study were obtained from Wave 5 data, such as past-month any tobacco use, cigarette use, e-cigarette use, and any other tobacco product use (ie, noncigarette and non–e-cigarette); nicotine dependence; and past-year methods used for smoking cessation. Past-month binge alcohol use (defined as an average of ≥5 drinks for males or ≥4 for females on days when alcohol was consumed in the past month), past-month cannabis and other substance use, and sociodemographic characteristics (ie, age, sex, race and ethnicity, educational level, and annual family income) were also from Wave 5 data. Race and ethnicity were self-identified by respondents and were based on the classifications developed by the US Census Bureau (eg, Hispanic, non-Hispanic Black [hereafter Black], non-Hispanic White [hereafter White], and non-Hispanic other [including American Indian or Alaska Native, Asian, Native Hawaiian or other Pacific Islander, and more than 1 race]).

Any tobacco included cigarettes, e-cigarettes, traditional cigars, cigarillos, filtered cigars, hookah, pipe tobacco, smokeless tobacco, loose snus or snus pouches, and dissolvable tobacco. Polycombustible and noncombustible tobacco included all combustible products (ie, cigarettes, any cigars, hookah, and pipe tobacco), e-cigarettes, and smokeless tobacco. Any cigars included traditional cigars, filtered cigars, and cigarillos. Any smokeless tobacco included smokeless tobacco and loose snus or snus pouches. Other substances included prescription medications that were not used as prescribed (eg, Ritalin, Adderall, painkillers, and sedatives or tranquilizers), cocaine or crack, stimulants (eg, methamphetamine or speed), and other drugs (eg, heroin, inhalants, solvent, and hallucinogens).

Past-year externalizing and internalizing problems (defined as validated measures of mental health disorders from the modified Global Appraisal of Individual Needs–Short Screener^28^) were assessed in Wave 5. Nicotine dependence scores were derived from responses to a 16-item questionnaire developed by Strong and colleagues^29^ that has demonstrated good validity and reliability.^29,30,31,32^

### Statistical Analysis

Data analyses were conducted between September 2021 and October 2022. First, exploratory, descriptive analyses were performed to examine differences in sociodemographic characteristics, behavioral health status, and distributions of types of tobacco products used (by those with past-month tobacco use) between individuals with and without lifetime psychosis. Pearson χ^2^ with Rao-Scott second-order correction for categorical variables and an unpaired, 2-tailed *t* test for continuous variables were used to test for statistical significance. Second, bivariable and multivariable logistic regressions (adjusting for differences resulting from the descriptive analyses) were applied to examine the differences between people with and without lifetime psychosis in unadjusted and adjusted past-month prevalence of any tobacco use, cigarette use, e-cigarette use, and any other tobacco product use overall and by sex, age, and race and ethnicity.

Third, among adults with past-month cigarette use, bivariable and multivariable linear regressions were conducted to identify the differences between those with and those without lifetime psychosis in unadjusted and adjusted mean nicotine dependence scores overall and by age, sex, and race and ethnicity. Bivariable and multivariable logistic regressions were applied to assess the differences in unadjusted and adjusted prevalence of smoking cessation methods used in the past year between individuals with and without lifetime psychosis. For each analysis, 2-tailed *P* < .05 was considered to be statistically significant. Stata, version 16 (StataCorp LLC) was used for all analyses.

## Results

We examined data on 29 045 civilian, noninstitutionalized adult participants in the Wave 5 survey of the PATH Study (eFigure in Supplement 1). These individuals had a weighted median (IQR) age of 30.0 (22.0-50.0) years and included 14 976 females (51.5%) and 14 069 males (48.5%), of whom 11.1% had Black, 16.0% had Hispanic, 65.0% had White, and 8.0% had non-Hispanic other race and ethnicity (weighted percentage estimates) (Table 1).

**Table 1.** Sociodemographic Characteristics and Behavioral Health Status Among Community-Dwelling Adults With vs Without Lifetime Psychosis

	Weighted % (SE)		*P* value
	With lifetime psychosis (n = 1186)	Without lifetime psychosis (n = 27 859)	
Overall	2.9 (0.1)	97.1 (0.1)	NA
Mean (SD) age, y	41.1 (0.7)	47.4 (0.1)	<.001
Age, y			
18-20	5.7 (0.5)	4.9 (0.1)	<.001
21-24	9.8 (1.0)	6.9 (0.1)	
25-44	43.5 (1.8)	34.4 (0.3)	
≥45	41.0 (2.2)	53.8 (0.3)	
Sex			
Male	45.4 (2.0)	48.6 (0.2)	.07
Female	54.6 (2.0)	51.5 (0.2)	
Race and ethnicity^a^			
Black	18.7 (1.6)	11.1 (0.1)	<.001
Hispanic	14.0 (1.6)	16.0 (0.1)	
White	60.5 (1.9)	65.0 (0.2)	
Other^b^	6.8 (1.0)	8.0 (0.1)	
Educational level			
<High school	13.6 (1.1)	9.4 (0.2)	<.001
High school diploma or equivalent	33.5 (1.7)	27.1 (0.3)	
Some college or associate’s degree	34.9 (2.1)	31.6 (0.3)	
≥Bachelor’s degree	18.0 (2.1)	32.0 (0.2)	
Annual family income, US $			
<25 000	58.8 (2.4)	26.1 (0.4)	<.001
25 000-74 999	29.7 (2.5)	38.5 (0.4)	
≥75 000	11.5 (1.5)	35.4 (0.5)	
Past-month binge alcohol use (yes)^c^	11.5 (1.2)	7.2 (0.2)	<.001
Past-month cannabis use (yes)	33.3 (2.1)	12.9 (0.3)	<.001
Past-month other substance use (yes)^d^	18.5 (1.6)	5.5 (0.2)	<.001
Past-year externalizing problems			
Low	35.4 (2.3)	70.3 (0.4)	<.001
Moderate	28.9 (2.1)	19.7 (0.4)	
High	35.7 (2.1)	10.0 (0.2)	
Past-year internalizing problems			
Low	22.9 (2.2)	67.3 (0.4)	<.001
Moderate	23.6 (1.9)	20.4 (0.4)	
High	53.6 (2.3)	12.3 (0.3)	

Abbreviation: NA, not applicable.

^a^ Race and ethnicity were self-identified by respondents in the PATH (Population Assessment of Tobacco and Health) Study based on the classifications developed by the US Census Bureau.

^b^ Non-Hispanic other race and ethnicity included American Indian or Alaska Native, Asian, Native Hawaiian and other Pacific Islander, and more than 1 race.

^c^ Binge alcohol use was defined as consumption of an average of 5 or more drinks for males and 4 or more drinks for females on days when alcohol was consumed in the past month.

^d^ Included Ritalin, Adderall, painkillers, and sedatives or tranquilizers that were not used as prescribed; cocaine or crack; stimulants, such as methamphetamine or speed; and other drugs such as heroin, inhalants, solvent, or hallucinogens.

Among these adults, 2.9% (95% CI, 2.62%-3.10%) had a lifetime diagnosis of psychosis (n = 1186) (Table 1). Compared with those without lifetime psychosis, individuals with lifetime psychosis were more likely to be aged 21 to 44 years, to be Black individuals, to have an annual family income of $25 000 or less, to use cannabis or other illicit substances in the past month, and to have high internalizing and externalizing problems in the past year. They were less likely to be 45 years or older, to have a bachelor’s degree or higher educational level, and to have an annual family income of $75 000 or more. Compared with those without psychosis, individuals with psychosis had a higher past-month prevalence of binge alcohol use (11.5% vs 7.2%; *P* < .001), cannabis use (33.3% vs 12.9%; *P* < .001), and other substance use (18.5 vs 5.5%; *P* < .001) (Table 1).

Among individuals with past-month tobacco use (Figure), the distributions of types of tobacco products used varied by lifetime psychosis status. Adults with lifetime psychosis (vs those without) had a higher prevalence of dual cigarette and e-cigarette use (13.5% vs 10.1%; *P* = .02), polycombustible tobacco use (12.1% vs 8.6%; *P* = .007), and polycombustible and noncombustible tobacco use (22.1% vs 12.4%; *P* < .001), but they had a lower prevalence of exclusive cigarette use (38.1% vs 42.5%; *P* = .02), exclusive e-cigarette use (6.4% vs 11.2%; *P* < .001), and exclusive any smokeless tobacco use (2.0% vs 5.6%; *P* < .001).

**Figure.**
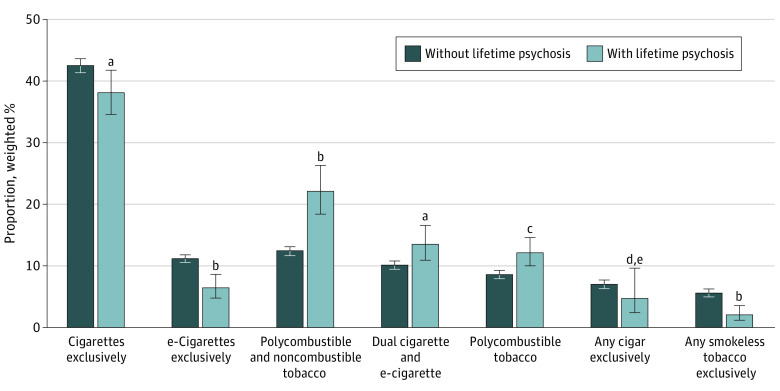
Tobacco Product Type Among Adults With Tobacco Use in Past Month Error bars represent 95% CIs. ^a^Difference between individuals with and without psychosis was significant (*P* = .02). ^b^Difference between individuals with and without psychosis was significant (*P* < .001). ^c^Difference between individuals with and without psychosis was significant (*P* = .007). ^d^Difference between individuals with and without psychosis was not significant (*P* = .23). ^e^Estimate should be interpreted with caution due to low statistical precision.

Adults with lifetime psychosis had a higher prevalence than adults without lifetime psychosis in the 4 tobacco use outcomes overall and in most subgroups. Compared with those without psychosis (Table 2), adults with lifetime psychosis had a higher adjusted past-month prevalence of any tobacco product use overall (41.3% vs 27.7%; adjusted risk ratio [RR], 1.49 [95% CI, 1.36-1.63]) and within each age, sex, and racial and ethnic group. Similarly, adults with lifetime psychosis had a higher adjusted prevalence of past-month cigarette smoking overall (30.7% vs 19.0%; adjusted RR, 1.61 [95% CI, 1.44-1.79]) and within each subgroup, but there was no difference in prevalence among individuals with or without lifetime psychosis in the non-Hispanic other race and ethnicity group (Table 2).

**Table 2.** Past-Month Prevalence of Tobacco Use by Product Type Among Community-Dwelling Adults With vs Without Lifetime Psychosis^a^

	Any tobacco use^b^			Cigarette use			e-Cigarette use			Any other tobacco product use^c^		
	With lifetime psychosis, weighted adjusted % (SE)	Without lifetime psychosis, weighted adjusted % (SE)	Adjusted RR (95% CI)	With lifetime psychosis, weighted adjusted % (SE)	Without lifetime psychosis, weighted adjusted % (SE)	Adjusted RR (95% CI)	With lifetime psychosis, weighted adjusted % (SE)	Without lifetime psychosis, weighted adjusted % (SE)	Adjusted RR (95% CI)	With lifetime psychosis, weighted adjusted % (SE)	Without lifetime psychosis, weighted adjusted % (SE)	Adjusted RR (95% CI)
Overall	41.3 (1.9)	27.7 (0.3)	1.49 (1.36-1.63)	30.7 (1.7)	19.0 (0.3)	1.61 (1.44-1.79)	13.3 (1.1)	8.8 (0.1)	1.51 (1.27-1.74)	16.7 (1.3)	10.2 (0.2)	1.65 (1.40-1.89)
Unweighted sample size	1186	27 845		1186	27 857		1186	27 852		1186	27 841	
**Age, y**												
18-20	43.3 (6.2)	31.3 (2.6)	1.38 (1.11-1.65)	18.6 (3.8)	10.0 (1.6)	1.86 (1.30-2.43)	33.4 (5.2)	26.1 (2.5)	1.28 (0.98-1.57)	25.6 (5.6)	13.6 (2.5)	1.87 (1.35-2.40)
Unweighted sample size	163	4949		163	4951		163	4950		163	4950	
21-24	43.0 (4.6)	33.4 (1.0)	1.29 (1.03-1.55)	24.6 (3.7)	16.7 (0.8)	1.47 (1.04-1.91)	24.0 (3.6)	18.8 (0.7)	1.28 (0.92-1.63)	22.3 (3.5)	14.8 (0.7)	1.51 (1.06-1.97)
Unweighted sample size	180	4530		180	4534		180	4533		180	4529	
25-44	49.5 (3.8)	34.8 (0.7)	1.42 (1.20-1.64)	35.6 (2.4)	25.4 (0.5)	1.40 (1.22-1.59)	15.7 (1.7)	10.9 (0.3)	1.44 (1.14-1.74)	19.7 (3.0)	12.8 (0.4)	1.54 (1.09-1.99)
Unweighted sample size	494	9745		494	9750		494	9749		494	9746	
≥45	37.5 (3.4)	23.7 (0.5)	1.59 (1.30-1.87)	32.0 (2.9)	18.1 (0.4)	1.77 (1.45-2.09)	7.87 (1.5)	4.1 (0.2)	1.92 (1.16-2.68)	14.1 (2.0)	7.5 (0.3)	1.86 (1.34-2.38)
Unweighted sample size	349	8621		349	8622		349	8620		349	8616	
**Sex**												
Male	47.6 (2.9)	32.4 (0.5)	1.47 (1.30-1.64)	31.8 (2.7)	20.7 (0.4)	1.54 (1.28-1.80)	16.3 (1.9)	9.5 (0.2)	1.72 (1.33-2.11)	25.6 (2.9)	14.9 (0.4)	1.72 (1.35-2.09)
Unweighted sample size	491	13 571		491	13 577		491	13 574		491	13 568	
Female	34.7 (2.5)	23.2 (0.4)	1.50 (1.28-1.71)	29.1 (2.3)	17.4 (0.4)	1.67 (1.40-1.94)	10.4 (1.0)	8.2 (0.2)	1.27 (1.02-1.51)	7.9 (0.7)	5.5 (0.2)	1.43 (1.16-1.70)
Unweighted sample size	695	14 274		695	14 280		695	14 278		695	14 273	
**Race and ethnicity^d^**												
Black	36.8 (3.2)	29.7 (0.8)	1.24 (1.02-1.45)	28.4 (2.8)	19.4 (0.7)	1.46 (1.18-1.75)	13.0 (2.4)	7.2 (0.5)	1.80 (1.18-2.43)	18.1 (2.0)	15.0 (0.7)	1.21 (0.95-1.47)
Unweighted sample size	238	4083		238	4085		238	4086		238	4084	
Hispanic	47.5 (7.3)	23.9 (0.9)	1.99 (1.40-2.58)	25.8 (3.7)	15.7 (0.7)	1.65 (1.16-2.13)	11.4 (2.4)	8.1 (0.4)	1.41 (0.83-1.99)	23.8 (6.5)	9.1 (0.6)	2.60 (1.25-3.95)
Unweighted sample size	193	5938		193	5944		193	5943		193	5938	
White	43.5 (2.6)	30.4 (0.5)	1.43 (1.26-1.60)	34.3 (2.5)	21.3 (0.4)	1.61 (1.39-1.84)	14.4 (1.4)	9.9 (0.2)	1.45 (1.16-1.74)	15.9 (1.5)	9.7 (0.3)	1.64 (1.34-1.93)
Unweighted sample size	644	15 674		644	15 677		644	15 672		644	15 671	
Other^e^	35.0 (4.7)	24.8 (1.4)	1.41 (1.05-1.77)	27.2 (4.5)	18.2 (1.2)	1.50 (0.99-2.01)	11.4 (2.8)	8.5 (0.6)	1.34 (0.66-2.02)	16.9 (3.8)	9.6 (0.8)	1.76 (0.94-2.57)
Unweighted sample size	111	2150		111	2151		111	2151		111	2148	

Abbreviation: RR, risk ratio.

^a^ Estimates adjusted for age, sex, race and ethnicity, educational level, annual family income, problematic alcohol use, cannabis and other substance use, and internalizing and externalizing problems (ie, validated measures of mental health disorders from the modified Global Appraisal of Individual Needs–Short Screener^28^) and should be interpreted with caution due to low statistical precision. Other substances: Ritalin, Adderall, painkillers, and sedatives or tranquilizers that were not used as prescribed; cocaine or crack; stimulants (eg, methamphetamine or speed); and other drugs (eg, heroin, inhalants, solvent, or hallucinogens).

^b^ Cigarettes, e-cigarettes, traditional cigars, cigarillos, filtered cigars, hookah, pipe tobacco, smokeless tobacco, loose snus or snus pouches, and dissolvable tobacco.

^c^ Noncigarette and non–e-cigarette.

^d^ Self-identified by respondents in PATH (Population Assessment of Tobacco and Health) Study based on US Census Bureau classifications.

^e^ American Indian or Alaska Native, Asian, Native Hawaiian and other Pacific Islander, and more than 1 race.

Compared with adults without lifetime psychosis (Table 2), adults with lifetime psychosis had a higher adjusted past-month prevalence of e-cigarette use overall (13.3% vs 8.8%; adjusted RR, 1.51 [95% CI, 1.27-1.74]) and in most of the subgroups; however, there was no difference between those with and without psychosis in the 18-to-24-year, non-Hispanic other race and ethnicity, or Hispanic groups. Compared with adults without lifetime psychosis (Table 2), adults with lifetime psychosis had a higher adjusted past-month prevalence of any other tobacco product use overall (16.7% vs 10.2%; adjusted RR, 1.65 [95% CI, 1.40-1.89]) and in most subgroups, but there was no difference between those with and without psychosis in the Black or non-Hispanic other race and ethnicity group.

Compared with their counterparts without lifetime psychosis, adults with lifetime psychosis and past-month cigarette use had higher adjusted mean nicotine dependence scores overall (54.6 [95% CI, 51.9-57.4] vs 49.5 [95% CI, 48.6-50.3]; *P* < .001) and within the 45-year-or-older (61.7 [95% CI, 57.6-65.8] vs 54.9 [95% CI, 53.6-56.2]; *P* = .002), female (56.9 [95% CI, 52.7-61.1] vs 49.8 [95% CI, 48.5-51.2]; *P* = .001), and Hispanic (53.7 [95% CI, 44.2-63.2] vs 40.0 [95% CI, 36.8-43.1]; *P* = .01) and Black (53.4 [95% CI, 48.7-58.1] vs 46.0 [95% CI, 43.9-48.1]; *P* = .005) groups (Table 3). Nicotine dependence severity did not vary by lifetime psychosis status among those aged 18 to 44 years, males, and White adults.

**Table 3.** Mean Nicotine Dependence Score Among Community-Dwelling Adults With vs Without Lifetime Psychosis Who Smoked Cigarettes in Past Month

	Weighted Mean Nicotine Dependence Score (95% CI)				Difference in mean score between groups			
	With lifetime psychosis		Without lifetime psychosis					
	Unadjusted	Adjusted^a^	Unadjusted	Adjusted^a^	Unadjusted (95% CI)	*P* value	Adjusted (95% CI)^a^	*P* value
Overall	62.5 (59.7 to 65.3)	54.6 (51.9 to 57.4)	50.6 (49.6 to 51.6)	49.5 (48.6 to 50.3)	11.9 (8.9 to 14.9)	.001	5.2 (2.3 to 8.0)	.001
Unweighted sample size	586	586	6478	6478				
**Age, y**								
18-24	51.2 (43.2 to 59.1)	40.8 (32.0 to 49.7)	39.6 (37.3 to 41.8)	35.5 (32.7 to 38.3)	11.6 (3.0 to 20.2)	.009	5.4 (−3.3 to 14.0)	.22
Unweighted sample size	88	88	829	829				
25-44	60.2 (56.2 to 64.2)	51.1 (47.0 to 55.1)	48.3 (47.1 to 49.5)	47.5 (46.5 to 48.5)	11.9 (7.8 to 15.9)	<.001	3.5 (−0.5 to 7.5)	.09
Unweighted sample size	275	275	2909	2909				
≥45	67.4 (63.6 to 71.2)	61.7 (57.6 to 65.8)	54.3 (52.9 to 55.8)	54.9 (53.6 to 56.2)	13.1 (8.9 to 17.2)	<.001	6.9 (2.6 to 11.1)	.002
Unweighted sample size	223	223	2740	2740				
**Sex**								
Male	59.4 (55.0 to 63.9)	52.1 (47.6 to 56.6)	49.4 (48.1 to 50.7)	49.2 (48.0 to 50.3)	10.0 (5.4 to 14.6)	<.001	2.9 (−1.7 to 7.6)	.21
Unweighted sample size	228	228	3139	3139				
Female	64.8 (60.9 to 68.6)	56.9 (52.7 to 61.1)	52.0 (50.7 to 53.3)	49.8 (48.5 to 51.2)	12.8 (8.6 to 16.9)	<.001	7.1 (2.9 to 11.2)	.001
Unweighted sample size	358	358	3339	3339				
**Race and ethnicity^b^**								
Black	58.2 (53.8 to 62.6)	53.4 (48.7 to 58.1)	45.1 (43.2 to 47.0)	46.0 (43.9 to 48.1)	13.2 (8.5 to 17.9)	<.001	7.4 (2.3 to 12.4)	.005
Unweighted sample size	106	106	1002	1002				
Hispanic	61.4 (52.5 to 70.4)	53.7 (44.2 to 63.2)	40.5 (37.7 to 43.4)	40.0 (36.8 to 43.1)	20.9 (11.1 to 30.6)	<.001	13.8 (3.4 to 24.1)	.01
Unweighted sample size	74	74	915	915				
White	63.7 (59.9 to 67.5)	55.4 (51.7 to 59.1)	54.0 (52.7 to 55.3)	52.6 (51.4 to 53.7)	9.7 (5.7 to 13.7)	<.001	2.9 (−1.0 to 6.7)	.14
Unweighted sample size	355	355	4104	4104				
Other^c^	62.2 (52.8 to 71.5)	54.6 (46.0 to 63.3)	46.7 (42.9 to 50.4)	47.1 (43.8 to 50.4)	15.5 (5.8 to 25.3)	.002	7.5 (−1.1 to 16.2)	.09
Unweighted sample size	51	51	457	457				

^a^ Estimates were adjusted for age, sex, race and ethnicity, educational level, annual family income, problematic alcohol use, cannabis and other substance use, and internalizing and externalizing problems (ie, validated measures of mental health disorders from the modified Global Appraisal of Individual Needs–Short Screener^28^). Other substances: Ritalin, Adderall, painkillers, and sedatives or tranquilizers that were not used as prescribed; cocaine or crack; stimulants, such as methamphetamine or speed; and other drugs such as heroin, inhalants, solvent, or hallucinogens.

^b^ Self-identified by respondents in the PATH (Population Assessment of Tobacco and Health) Study based on US Census Bureau classifications.

^c^ American Indian or Alaska Native, Asian, Native Hawaiian or other Pacific Islander, and more than 1 race.

Among adults with past-month cigarette smoking, those with vs without lifetime psychosis were more likely to make a quit attempt (adjusted weighted prevalence: 60.0% vs 54.1%; adjusted RR, 1.11 [95% CI, 1.01-1.21]) (Table 4) and to use any tobacco cessation approach such as e-cigarettes; NRT; prescription medications; or counseling, a quitline, a support group, or a web-based program (17.8% vs 13.3%; adjusted RR, 1.35 [95% CI, 1.00-1.70]). Adults with lifetime psychosis were 2.3 times more likely than those without psychosis to use counseling, a quitline, or a support group for tobacco cessation (5.6% vs 2.5%; adjusted RR, 2.25 [95% CI, 1.21-3.30]). Adjusted past-year prevalence of using e-cigarettes, NRT, and prescription tobacco cessation medications was similar between the 2 populations.

**Table 4.** Prevalence of Smoking Cessation Methods Used in Past 12 Months Among Community-Dwelling Adults With vs Without Lifetime Psychosis Who Smoked Cigarettes in Past Month

Strategies used to aid quitting in the past 12 mo	Adjusted weighted % (95% CI)		Unadjusted RR (95% CI)	Adjusted RR^a^ (95% CI)
	With lifetime psychosis (n = 584)	Without lifetime psychosis (n = 6457)		
Used e-cigarettes or other electronic nicotine products	5.1 (3.0-7.2)	4.6 (4.0-5.3)	1.53 (0.92-2.14)	1.10 (0.63-1.57)
Used nicotine replacement therapy^b^	8.7 (5.5-11.8)	6.3 (5.6-7.1)	1.65 (1.08-2.22)	1.37 (0.87-1.87)
Used prescription tobacco cessation medications^c^	4.5 (2.5-6.4)	3.6 (2.9-4.2)	1.36 (0.82-1.90)	1.26 (0.70-1.81)
Used counseling, quitline, support group, web-based program^d^	5.6 (3.1-8.0)	2.5 (2.0-3.0)	2.86 (1.77-3.96)	2.25 (1.21-3.30)
Used any of the above	17.8 (13.4-22.3)	13.3 (12.2-14.3)	1.63 (1.27-1.99)	1.35 (1.00-1.70)
Made a quit attempt in past year	60.0 (54.4-65.7)	54.1 (52.5-55.7)	1.19 (1.10-1.28)	1.11 (1.01-1.21)

Abbreviation: RR, risk ratio.

^a^ Adjusted for age, sex, race and ethnicity, educational level, annual family income, binge alcohol use, cannabis and other substance use, and internalizing and externalizing problems (ie, validated measures of mental health disorders from the modified Global Appraisal of Individual Needs–Short Screener^28^). Other substances included Ritalin, Adderall, painkillers, and sedatives or tranquilizers that were not used as prescribed; cocaine or crack; stimulants, such as methamphetamine or speed; and other drugs such as heroin, inhalants, solvent, or hallucinogens.

^b^ Nicotine patch, gum, inhaler, nasal spray, and lozenge.

^c^ Chantix, varenicline, Wellbutrin, Zyban, and bupropion.

^d^ Counseling; telephone helpline or quitline; books, pamphlets, and videos; tobacco cessation clinic, class, or support group; and online or web-based program.

## Discussion

Among community-dwelling adults with lifetime psychosis in the US between 2018 and 2019, the past-month prevalence was 41.3% for any tobacco use and was 30.7% for cigarette smoking. By contrast, an earlier study using 2004 to 2005 nationally representative data found that past-month cigarette smoking prevalence was 49.8% among US adults with lifetime psychosis.^5^ These differences are consistent with a recent population-based study finding of a significant increase in smoking cessation for adults with psychiatric conditions.^8^

Compared with adults without psychosis, those with psychosis had a 1.5- to 1.6-times higher adjusted prevalence of past-month any tobacco use and past-month cigarette smoking overall; had a 1.2- to 2.0-times higher prevalence within almost every age, sex, and racial and ethnic group; and had a higher adjusted prevalence of e-cigarette use and any other tobacco product use overall and in most subgroups. A recently proposed bidirectional association that suggested smoking may be causally associated with an elevated risk of psychosis through shared genetic liability to smoking and psychosis^33,34^ may help explain the present study’s findings. Moreover, people with psychosis may seek nicotine to alleviate the symptoms of their illness or the adverse effects of antipsychotic medications.^34^

Although cigarettes remain the most common type of tobacco product used among adults with psychosis (as in the overall US population), the high prevalence of polytobacco product use in this population indicates the need for clinical efforts to address all types of tobacco use. In the general adult population, polytobacco use has been associated with a greater likelihood of nicotine dependence symptoms than single tobacco product use.^35^ We found that, among adults with past-month cigarette use, those with lifetime psychosis had greater polytobacco use and higher adjusted mean nicotine dependence scores than those without psychosis. Results of the present study are consistent with those of a small study (n = 40) that found people with schizophrenia smoked more intensely than their matched nonpsychiatric counterparts^36^ and findings of a clinical study (n = 613) that reported a high smoking prevalence and nicotine dependence severity from early through late psychosis.^37^ In accordance with these findings, a recent study also found overlapping neural mechanisms of nicotine dependence and psychosis.^38^

Tobacco consumption has been associated with higher levels of psychosis symptoms and lower cognitive function,^39,40^ and greater smoking severity was a factor in increased premature mortality risk.^41^ By contrast, tobacco cessation among people with psychosis has been associated with improved cognitive function and psychosis symptoms^42,43^and with reduced morbidity.^35^ Yet a systematic literature review has shown that people with schizophrenia and tobacco use are less likely to receive tobacco cessation support from clinicians than their counterparts without schizophrenia.^44^

In addition to addressing disparities in treatments for psychosis,^16,45^ clinicians can play an important role in smoking cessation for individuals with psychosis. Health concerns were the most cited facilitator in quitting smoking among people with schizophrenia.^44^ In the present study, adults with psychosis were more likely to make a quit attempt than adults without psychosis. Among adults with past-month cigarette smoking and psychosis, almost two-thirds made a quit attempt in the past year and 17.8% used a tobacco cessation approach. These findings suggest that past-year prevalence of using a specific tobacco cessation approach was not lower among adults with psychosis who smoke cigarettes than among their counterparts without psychosis, which is consistent with the results of a clinical trial^46^ that demonstrated that individuals with or without psychiatric diagnoses attempted to quit smoking at similar rates.^46^ We conclude that targeted smoking cessation strategies need to be part of the comprehensive management of psychosis.

Given that higher nicotine dependence severity is associated with smoking relapse,^47,48^ future research is needed to develop smoking cessation interventions that are tailored for people with psychosis who have had multiple quit attempts and smoking relapses. Evidence-based; age, sex, and race and ethnicity appropriate; and targeted smoking cessation strategies need to be integrated into psychosis management, especially for individuals 45 years or older, female individuals, and Hispanic and Black adults with high nicotine dependence severity.

Furthermore, the analyses uncovered a much higher frequency of past-month use of cannabis and other substances among adults with a history of psychosis than in those without psychosis. Even when the analyses adjusted for differences in these measures, other behavioral conditions, and sociodemographic characteristics, tobacco use remained significantly associated with psychosis. Further research is needed to examine the interactions between nicotine and cannabis consumption and to evaluate strategies for smoking cessation in individuals with comorbid use of other substances.

This study found that compared with their counterparts without psychosis, community-dwelling individuals in the US with lifetime psychosis were more likely to be aged 25 to 44 years, to have low annual income, to be Black adults, and to have substance use and comorbid mental health conditions but were less likely to be aged 45 years or older. These results are consistent with the recognition that smoking in individuals with psychosis is associated with premature mortality and an overlooked health inequity, especially for Black individuals.^16,49^ Lower income and educational level among persons with psychosis suggest that resilient environments and coping resources for people with psychosis may be limited, and such inadequacies may have been further exacerbated during the COVID-19 pandemic. Future research should examine changes in tobacco use among people with or without psychosis before and during the COVID-19 pandemic.

### Limitations

This study has several limitations. First, the cross-sectional analysis of the PATH Study data precluded drawing causal associations. Although the PATH Study has longitudinal data, psychosis measures were collected only in recent waves, and access to longitudinal data is needed to examine these measures. A follow-up study may be conducted using these longitudinal data when future data are available and if sample sizes are sufficient. Second, the PATH Study is a self-reported survey and is subject to recall and social desirability biases. For example, measures of psychosis were based on self-reported diagnoses from clinicians and did not distinguish between acute and chronic psychosis. We estimated a 2.9% diagnosed lifetime psychosis prevalence because psychosis is often underdiagnosed and misdiagnosed^50^ and because people may not recognize their own symptoms as psychosis. We may have underestimated the differences in outcomes between individuals with and without psychosis. Third, the variability in differences between adults with and without psychosis across subgroups may be due to sample sizes and power considerations, although the differences in most cases appeared consistent, even if not statistically significant. Fourth, the PATH Study did not assess the quantity and quality of tobacco cessation approaches used by individuals with or without psychosis and the timing and intensity of receipt of mental health care among those with psychosis.

## Conclusions

In this cross-sectional study, a high prevalence of tobacco use, polytobacco use, and making a quit attempt and high nicotine dependence severity highlighted the urgent need for tailoring tobacco cessation interventions for persons with psychosis. Evidence-based; age, sex, and race and ethnicity appropriate; and targeted tobacco cessation strategies need to be part of a comprehensive medical treatment of psychosis.

## Notes

Supplement 1. **eFigure.** Flowchart

Supplement 2.
Data Sharing Statement

## References

[1] Siskind DJ, Wu BT, Wong TT, Firth J, Kisely S. Pharmacological interventions for smoking cessation among people with schizophrenia spectrum disorders: a systematic review, meta-analysis, and network meta-analysis. Lancet Psychiatry. 2020;7(9):762-774. doi:10.1016/S2215-0366(20)30261-3 32828166

[2] Fusar-Poli P, Salazar de Pablo G, Correll CU, . Prevention of psychosis: advances in detection, prognosis, and intervention. JAMA Psychiatry. 2020;77(7):755-765. doi:10.1001/jamapsychiatry.2019.4779 32159746

[3] Gurillo P, Jauhar S, Murray RM, MacCabe JH. Does tobacco use cause psychosis? systematic review and meta-analysis. Lancet Psychiatry. 2015;2(8):718-725. doi:10.1016/S2215-0366(15)00152-2 26249303 PMC4698800

[4] Anthenelli RM, Benowitz NL, West R, . Neuropsychiatric safety and efficacy of varenicline, bupropion, and nicotine patch in smokers with and without psychiatric disorders (EAGLES): a double-blind, randomised, placebo-controlled clinical trial. Lancet. 2016;387(10037):2507-2520. doi:10.1016/S0140-6736(16)30272-0 27116918

[5] Smith PH, Mazure CM, McKee SA. Smoking and mental illness in the U.S. population. Tob Control. 2014;23(e2):e147-e153. doi:10.1136/tobaccocontrol-2013-051466 24727731 PMC4201650

[6] Caponnetto P, Polosa R. Approved and emerging smoking cessation treatments for people with schizophrenia spectrum disorders: a narrative review. Health Psychol Res. 2020;8(2):9237. doi:10.4081/hpr.2020.9237 33123649 PMC7588850

[7] Wong JA, Pratt SI, Ferron JC, Gowarty M, Brunette MF. Characteristics of and reasons for electronic cigarette use among adult smokers with schizophrenia/schizoaffective disorder. Ann Clin Psychiatry. 2022;34(1):89-96. doi:10.12788/acp.0050 35166668

[8] Han B, Volkow ND, Blanco C, Tipperman D, Einstein EB, Compton WM. Trends in prevalence of cigarette smoking among US adults with major depression or substance use disorders, 2006-2019. JAMA. 2022;327(16):1566-1576. doi:10.1001/jama.2022.4790 35471512 PMC9044114

[9] McGrath JJ, Saha S, Al-Hamzawi A, . Psychotic experiences in the general population: a cross-national analysis based on 31,261 respondents from 18 countries. JAMA Psychiatry. 2015;72(7):697-705. doi:10.1001/jamapsychiatry.2015.0575 26018466 PMC5120396

[10] US Department of Health and Human Services. Smoking Cessation: A Report of the Surgeon General. U.S. Department of Health and Human Services, Centers for Disease Control and Prevention, National Center for Chronic Disease Prevention and Health Promotion, Office on Smoking and Health; 2020.

[11] Ho RKS, Lee GMT, Fok PWY, Chan HCH, Ching JKW. Characteristics of Chinese smokers with psychotic disorders and their predictors on smoking cessation in Hong Kong. Tob Prev Cessat. 2020;6:7. doi:10.18332/tpc/115030 32548344 PMC7291916

[12] Solty H, Crockford D, White WD, Currie S. Cigarette smoking, nicotine dependence, and motivation for smoking cessation in psychiatric inpatients. Can J Psychiatry. 2009;54(1):36-45. doi:10.1177/070674370905400107 19175978

[13] Han B. *Key Substance Use and Mental Health Indicators in the United States: Results From the 2019 National Survey on Drug Use and Health*. HHS Publication No. PEP20-07-01-001, NSDUH Series H-55. Substance Abuse and Mental Health Services Administration; 2020.

[14] Aleman A, Kahn RS, Selten JP. Sex differences in the risk of schizophrenia: evidence from meta-analysis. Arch Gen Psychiatry. 2003;60(6):565-571. doi:10.1001/archpsyc.60.6.565 12796219

[15] Ochoa S, Usall J, Cobo J, Labad X, Kulkarni J. Gender differences in schizophrenia and first-episode psychosis: a comprehensive literature review. Schizophr Res Treatment. 2012;2012:916198. doi:10.1155/2012/916198 22966451 PMC3420456

[16] Misra S, Etkins OS, Yang LH, Williams DR. Structural racism and inequities in incidence, course of illness, and treatment of psychotic disorders among Black Americans. Am J Public Health. 2022;112(4):624-632. doi:10.2105/AJPH.2021.306631 35319958 PMC8961835

[17] Schoer N, Huang CW, Anderson KK. Differences in duration of untreated psychosis for racial and ethnic minority groups with first-episode psychosis: an updated systematic review and meta-analysis. Soc Psychiatry Psychiatr Epidemiol. 2019;54(10):1295-1298. doi:10.1007/s00127-019-01737-3 31183503

[18] Ryan JE, Veliz P, McCabe SE, Stoddard SA, Boyd CJ. Association of early onset of cannabis, cigarette, other drug use and schizophrenia or psychosis. Schizophr Res. 2020;215:482-484. doi:10.1016/j.schres.2019.10.002 31623965 PMC7035998

[19] National Addiction & HIV Data Archive Program. Population Assessment of Tobacco and Health (PATH) study series. Accessed June 10, 2022. https://www.icpsr.umich.edu/web/NAHDAP/series/606

[20] National Institute on Alcohol Abuse and Alcoholism. National Epidemiologic Survey on Alcohol and Related Conditions-III (NESARC-III). Accessed January 2, 2023. https://www.niaaa.nih.gov/research/nesarc-iii

[21] McMillan KA, Enns MW, Cox BJ, Sareen J. Comorbidity of axis I and II mental disorders with schizophrenia and psychotic disorders: findings from the National Epidemiologic Survey on Alcohol and Related Conditions. Can J Psychiatry. 2009;54(7):477-486. doi:10.1177/070674370905400709 19660170

[22] Davis GP, Compton MT, Wang S, Levin FR, Blanco C. Association between cannabis use, psychosis, and schizotypal personality disorder: findings from the National Epidemiologic Survey on Alcohol and Related Conditions. Schizophr Res. 2013;151(1-3):197-202. doi:10.1016/j.schres.2013.10.018 24200416 PMC3877688

[23] Nepon J, Belik SL, Bolton J, Sareen J. The relationship between anxiety disorders and suicide attempts: findings from the National Epidemiologic Survey on Alcohol and Related Conditions. Depress Anxiety. 2010;27(9):791-798. doi:10.1002/da.20674 20217852 PMC2940247

[24] Supina AL, Patten SB. Self-reported diagnoses of schizophrenia and psychotic disorders may be valuable for monitoring and surveillance. Can J Psychiatry. 2006;51(4):256-259. doi:10.1177/070674370605100407 16629350

[25] Jablensky A. The 100-year epidemiology of schizophrenia. Schizophr Res. 1997;28(2-3):111-125. doi:10.1016/S0920-9964(97)85354-6 9468347

[26] Mueser KT, McGurk SR. Schizophrenia. Lancet. 2004;363(9426):2063-2072. doi:10.1016/S0140-6736(04)16458-1 15207959

[27] Jablensky A, Sartorius N, Ernberg G, . Schizophrenia: manifestations, incidence and course in different cultures—a World Health Organization ten-country study. Psychol Med Monogr Suppl. 1992;20:1-97. doi:10.1017/S0264180100000904 1565705

[28] Dennis ML, Chan YF, Funk RR. Development and validation of the GAIN Short Screener (GSS) for internalizing, externalizing and substance use disorders and crime/violence problems among adolescents and adults. Am J Addict. 2006;15(suppl 1):80-91. doi:10.1080/10550490601006055 17182423 PMC5933850

[29] Strong DR, Leas E, Noble M, . Predictive validity of the adult tobacco dependence index: findings from waves 1 and 2 of the Population Assessment of Tobacco and Health (PATH) study. Drug Alcohol Depend. 2020;214:108134. doi:10.1016/j.drugalcdep.2020.108134 32629146 PMC7446939

[30] Snell M, Harless D, Shin S, Cunningham P, Barnes A. A longitudinal assessment of nicotine dependence, mental health, and attempts to quit smoking: evidence from waves 1-4 of the Population Assessment of Tobacco and Health (PATH) study. Addict Behav. 2021;115:106787. doi:10.1016/j.addbeh.2020.106787 33383566 PMC7837319

[31] Cwalina SN, Majmundar A, Unger JB, Barrington-Trimis JL, Pentz MA. Adolescent menthol cigarette use and risk of nicotine dependence: findings from the national Population Assessment on Tobacco and Health (PATH) study. Drug Alcohol Depend. 2020;206:107715. doi:10.1016/j.drugalcdep.2019.107715 31760252 PMC6980659

[32] Snell LM, Barnes AJ, Nicksic NE. A longitudinal analysis of nicotine dependence and transitions from dual use of cigarettes and electronic cigarettes: evidence from waves 1-3 of the PATH study. J Stud Alcohol Drugs. 2020;81(5):595-603. doi:10.15288/jsad.2020.81.595 33028472 PMC8076487

[33] King M, Jones R, Petersen I, Hamilton F, Nazareth I. Cigarette smoking as a risk factor for schizophrenia or all non-affective psychoses. Psychol Med. 2021;51(8):1373-1381. doi:10.1017/S0033291720000136 32148211

[34] Quigley H, MacCabe JH. The relationship between nicotine and psychosis. Ther Adv Psychopharmacol. Published online July 1, 2019. doi:10.1177/2045125319859969 31308936 PMC6604123

[35] Sung HY, Wang Y, Yao T, Lightwood J, Max W. Polytobacco use and nicotine dependence symptoms among US adults, 2012-2014. Nicotine Tob Res. 2018;20(suppl 1):S88-S98. doi:10.1093/ntr/nty050 30125019 PMC6093419

[36] Tidey JW, Rohsenow DJ, Kaplan GB, Swift RM. Cigarette smoking topography in smokers with schizophrenia and matched non-psychiatric controls. Drug Alcohol Depend. 2005;80(2):259-265. doi:10.1016/j.drugalcdep.2005.04.002 15869844

[37] Lally J, Spaducci G, Gardner-Sood P, . Tobacco smoking and nicotine dependence in first episode and established psychosis. Asian J Psychiatr. 2019;43:125-131. doi:10.1016/j.ajp.2019.05.002 31132542

[38] Schermitzler B, Miley K, Vinogradov S, Ramsay IS. Smoking is related to reduced motivation, but not global cognition, in the first two years of treatment for first episode psychosis. J Clin Med. 2021;10(8):1619. doi:10.3390/jcm10081619 33920376 PMC8069411

[39] van der Heijden HS, Schirmbeck F, McGuire P, ; EU-GEI High Risk Study. Association between tobacco use and symptomatology in individuals at ultra-high risk to develop a psychosis: a longitudinal study. Schizophr Res. 2021;236:48-53. doi:10.1016/j.schres.2021.08.006 34390981

[40] Ding JB, Hu K. Cigarette smoking and schizophrenia: etiology, clinical, pharmacological, and treatment implications. Schizophr Res Treatment. 2021;2021:7698030. doi:10.1155/2021/7698030 34938579 PMC8687814

[41] Kelly DL, McMahon RP, Wehring HJ, . Cigarette smoking and mortality risk in people with schizophrenia. Schizophr Bull. 2011;37(4):832-838. doi:10.1093/schbul/sbp152 20019128 PMC3122289

[42] Vermeulen JM, Schirmbeck F, Blankers M, ; Genetic Risk and Outcome of Psychosis (GROUP) Investigators. Association between smoking behavior and cognitive functioning in patients with psychosis, siblings, and healthy control subjects: results from a prospective 6-year follow-up study. Am J Psychiatry. 2018;175(11):1121-1128. doi:10.1176/appi.ajp.2018.18010069 30138044

[43] Baker A, Richmond R, Lewin TJ, Kay-Lambkin F. Cigarette smoking and psychosis: naturalistic follow up 4 years after an intervention trial. Aust N Z J Psychiatry. 2010;44(4):342-350. doi:10.3109/00048670903489841 20307166

[44] Lum A, Skelton E, Wynne O, Bonevski B. A systematic review of psychosocial barriers and facilitators to smoking cessation in people living with schizophrenia. Front Psychiatry. 2018;9:565. doi:10.3389/fpsyt.2018.00565 30459658 PMC6232499

[45] Oluwoye O, Stiles B, Monroe-DeVita M, . Racial-ethnic disparities in first-episode psychosis treatment outcomes from the RAISE-ETP study. Psychiatr Serv. 2018;69(11):1138-1145. doi:10.1176/appi.ps.201800067 30152275 PMC6395511

[46] Tulloch HE, Pipe AL, Clyde MJ, Reid RD, Els C. The quit experience and concerns of smokers with psychiatric illness. Am J Prev Med. 2016;50(6):709-718. doi:10.1016/j.amepre.2015.11.006 26711162

[47] Prochaska JJ, Benowitz NL. Current advances in research in treatment and recovery: nicotine addiction. Sci Adv. 2019;5(10):eaay9763. doi:10.1126/sciadv.aay9763 31663029 PMC6795520

[48] Grant BF, Shmulewitz D, Compton WM. Nicotine use and DSM-IV nicotine dependence in the United States, 2001–2002 and 2012–2013. Am J Psychiatry. 2020;177(11):1082-1090. doi:10.1176/appi.ajp.2020.19090900 32791895

[49] Heun-Johnson H, Menchine M, Axeen S, . Association between race/ethnicity and disparities in health care use before first-episode psychosis among privately insured young patients. JAMA Psychiatry. 2021;78(3):311-319. doi:10.1001/jamapsychiatry.2020.3995 33355626 PMC7758828

[50] Lieberman JA, Fenton WS. Delayed detection of psychosis: causes, consequences, and effect on public health. Am J Psychiatry. 2000;157(11):1727-1730. doi:10.1176/appi.ajp.157.11.1727 11058464

