# Supplementary material for: Tobacco Use, Nicotine Dependence, and Cessation Methods in US Adults With Psychosis
Source: JAMA Netw Open. 2023 Mar 28;6(3):e234995. doi: 10.1001/jamanetworkopen.2023.4995 (PMC10051107; doi:10.1001/jamanetworkopen.2023.4995)
Supplement: Supplement 1. — eFigure. Flowchart [file jamanetwopen-e234995-s001.pdf]

Supplemental Online Content

Han B, Aung TW, Volkow ND, et al. Tobacco use, nicotine dependence, and cessation methods in US adults with psychosis. *JAMA Netw Open*. 2023;6(3):e234995.
doi:10.1001/jamanetworkopen.2023.4995

eFigure. Flowchart

This supplemental material has been provided by the authors to give readers additional information about their work.

© 2023 Han B et al. *JAMA Network Open.*

eFigure. Flowchart

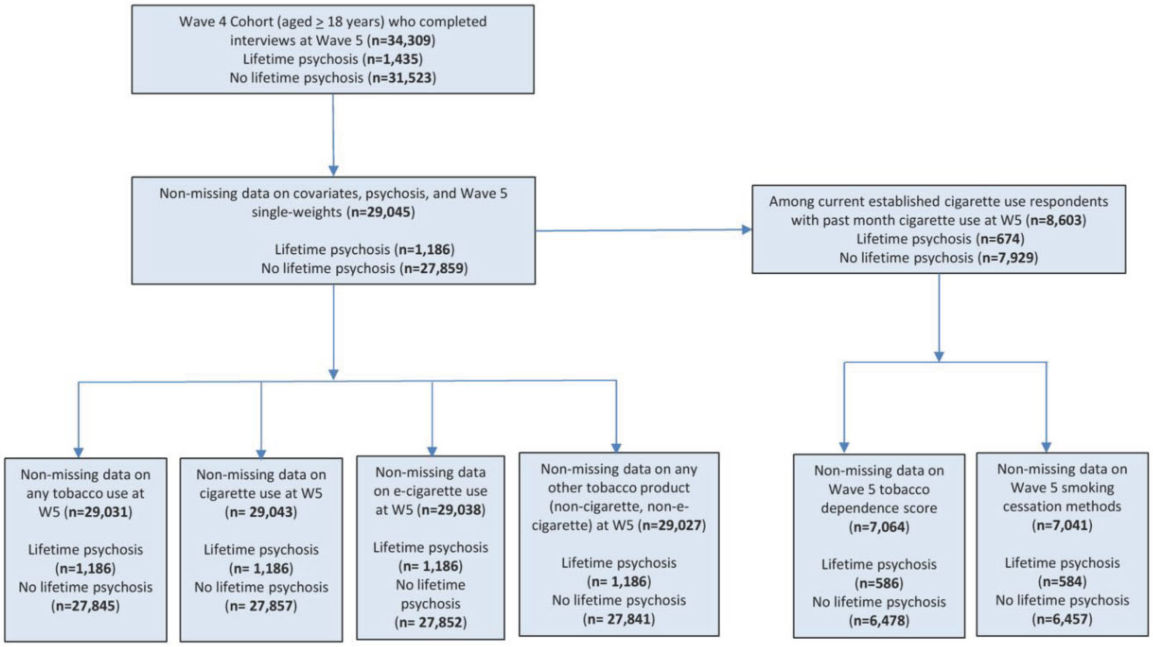

Flowchart depicting how the analytical samples of this study were derived

© 2023 Han B et al. *JAMA Network Open.*
