# Supplementary material for: Tobacco Use, Nicotine Dependence, and Cessation Methods in US Adults With Psychosis
Source: JAMA Netw Open. 2023 Mar 28;6(3):e234995. doi: 10.1001/jamanetworkopen.2023.4995 (PMC10051107; doi:10.1001/jamanetworkopen.2023.4995)
Supplement: Supplement 2. — Data Sharing Statement [file jamanetwopen-e234995-s002.pdf]

Data Sharing Statement

Han. Tobacco Use, Nicotine Dependence, and Cessation Methods in US Adults With Psychosis. *JAMA Netw Open*. Published March 28, 2023.
doi:10.1001/jamanetworkopen.2023.4995

Data
Data available: Yes
Data types: Deidentified participant data
How to access data: https://www.icpsr.umich.edu/web/NAHDAP/series/606
When available: With publication

Supporting Documents
Document types: None

Additional Information
Who can access the data: anyone requesting the data
Types of analyses: for any purpose
Mechanisms of data availability: without investigator support with a signed data access agreement
